# Poster Session I - A124PERCUTANEOUS TRANSHEPATIC BILIARY DRAINAGE (PTBD) FOR MALIGNANT HILAR BILIARY OBSTRUCTION (MHBO) IS ASSOCIATED WITH POOR OUTCOMES – IS IT TIME TO REASSESS ITS CLINICAL UTILITY?

**DOI:** 10.1093/jcag/gwaf042.124

**Published:** 2026-02-13

**Authors:** A Robertson, M Timmermans, G Buchanan, A Nordin-Garrett, S Perryman, P Mathura, D Bigam, J Shapiro, K Dajani, B Anderson, C Fung, J Nilsson, S Wasilenko, S Veldhuyzen Van Zanten, R Owen, G Sandha

**Affiliations:** University of Alberta College of Health Sciences, Edmonton, AB, Canada; Alberta Health Services, Edmonton, AB, Canada; Alberta Health Services, Edmonton, AB, Canada; Alberta Health Services, Edmonton, AB, Canada; Alberta Health Services, Edmonton, AB, Canada; Alberta Health Services, Edmonton, AB, Canada; University of Alberta College of Health Sciences, Edmonton, AB, Canada; University of Alberta College of Health Sciences, Edmonton, AB, Canada; University of Alberta College of Health Sciences, Edmonton, AB, Canada; University of Alberta College of Health Sciences, Edmonton, AB, Canada; University of Alberta College of Health Sciences, Edmonton, AB, Canada; University of Alberta College of Health Sciences, Edmonton, AB, Canada; University of Alberta College of Health Sciences, Edmonton, AB, Canada; Medicine, University of Alberta, Edmonton, NS, Canada; University of Alberta College of Health Sciences, Edmonton, AB, Canada; University of Alberta College of Health Sciences, Edmonton, AB, Canada

## Abstract

**Background:**

Decompression of malignant biliary obstruction is important for quality of life and potential chemotherapy (CTx). PTBD is an alternative when endoscopic stenting with ERCP is not possible. In MHBO, PTBD is the preferred intervention as multiple branches may be obstructed rendering ERCP stenting suboptimal.

**Aims:**

Assess the impact of PTBD for MHBO on patient outcomes and compare them to management of extrahepatic obstruction.

**Methods:**

Retrospective chart audit of all patients diagnosed with cholangiocarcinoma (CCA) at the University of Alberta Hospital from 07/2023-06/2024. Primary outcome was a reduction of the bilirubin to < 30 µmol/L. Secondary outcomes were successful initiation and completion of CTx, procedural complications, re-interventions, hospital length of stay (HLOS), and mortality.

**Results:**

30 pts (17 M), median age of 70±10 yrs (52-94 yrs) presented with CCA. Location of CCA was intrahepatic in 9, hilar in 13, and extrahepatic in 8 pts. No pts with intrahepatic CCA underwent PTBD and were not included in this review. We compared pts with MHBO and those with extrahepatic CCA (Table).

**Conclusions:**

The primary outcome was achieved with PTBD in 62% pts with MHBO but significant complications requiring re-intervention precluded optimal CTx, and were associated with a longer HLOS and shorter survival. Further inquiry into cost implications, and assessment of PTBD as standard of care in pts with MHBO is required.

**Funding Agencies:**

None

**Table:** 

	HILAR CCA	EXTRAHEPATIC CCA
Pts, n	13	8
Baseline bilirubin (bili) umol/L, median+/-SD	136+/-128	126+/-95
Intervention, n	-	-
None	2	2
ERCP only	1	6
PTBD only	6	0
ERCP->PTBD	3	0
PTBD->ERCP	1	0
Lowest post-intervention bili umol/L, median+/-SD	15+/-71	11+/-4
Time to lowest bili, days, median+/-SD	46+/-64	47+/-16
Primary outcome, n	-	-
Pts with bili <30 umol/L	8 (62%)	8 (100%)
Pts with bili >31 umol/L	5	0
Complications, n	-	-
Total	9/13 (69%)	3/8 (8%)
Bile leak	8	0
Drain/stent occlusion	7	2
Pancreatitis	1	1
Re-interventions, n	-	-
Pts needing repeat procedures	10/13 (77%)	4/8 (50%)
Total no. of repeat procedures	44 (range 1-11)	10 (range 1-5)
Overall rate of repeat procedures/pt	3.4	1.3
Chemotherapy, n	-	-
Pts referred cancer centre	11/13 (85%)	7/8 (88%)
Pts offered CTx	5/13 (38%)	6/8 (75%)
Pts that started CTx	3/13 (23%)	6/8 (75%)
Pts that finished CTx	0/13	2/8 (25%)
HLOS in days, mean+/-SD	22+/-16	7+/-7
Mortality, n	7 (54%)	5 (62%)
Survival post-intervention, days, mean+/-SD	158+/-102	286+/-41

